# Bilateral choanal atresia in an adult - management with mitomycin C and without stents: a case report

**DOI:** 10.1186/1757-1626-2-9307

**Published:** 2009-12-11

**Authors:** Fadlullah Aksoy, Hasan Demirhan, Yavuz Selim Yildirim, Orhan Ozturan

**Affiliations:** 1 Department of Otorhinolaryngology, Haseki Education and Research Hospital, Istanbul, Turkey

## Abstract

**Background:**

A 23-year-old female patient presented to the outpatient clinic with bilateral nasal obstruction and discharge since birth. Endoscopic examination and paranasal sinus tomography revealed bilateral choanal atresia. She did not have any other congenital abnormalities. Her parents reported cyanosis in childhood that worsened during feeding and improved during crying; however, they had not visited a physician. She did not have remarkable complaints during early childhood or adolescence.

**Methods:**

The patient was operated under general anesthesia, using a 0° 4 mm rigid endoscope. The orifice was widened with a curette and, to prevent stenosis, mitomycin-C (1 mg/ml) was applied topically to the nasopharyngeal orifice for 5 minutes. No stents were placed.

**Results:**

Follow-up evaluation at postoperative 12th month showed that her symptoms improved significantly and, on endoscopic examination, both choanae remained patent.

## Introduction

Choanal atresia was first described by Johann Roderer in 1755[1]. Congenital choanal atresia is unilateral or bilateral occlusion of the posterior nasal orifices. Being a rare abnormality, seen in 1 out of 5000-7000 live births [2], it is associated with other congenital abnormalities in 50% of the cases [3], and is twice as common in females. Seventy percent of the atresias are mixed bony-membranous type and 30% is pure bony type. The atresia may be complete or incomplete[4]. Bilateral choanal atresia is very rare in adults [3,5,6]. Newborns are obligatory nasal breathers, therefore bilateral choanal atresia is often diagnosed at birth and treated with early surgery.

## Case Report

**Figure 1:**
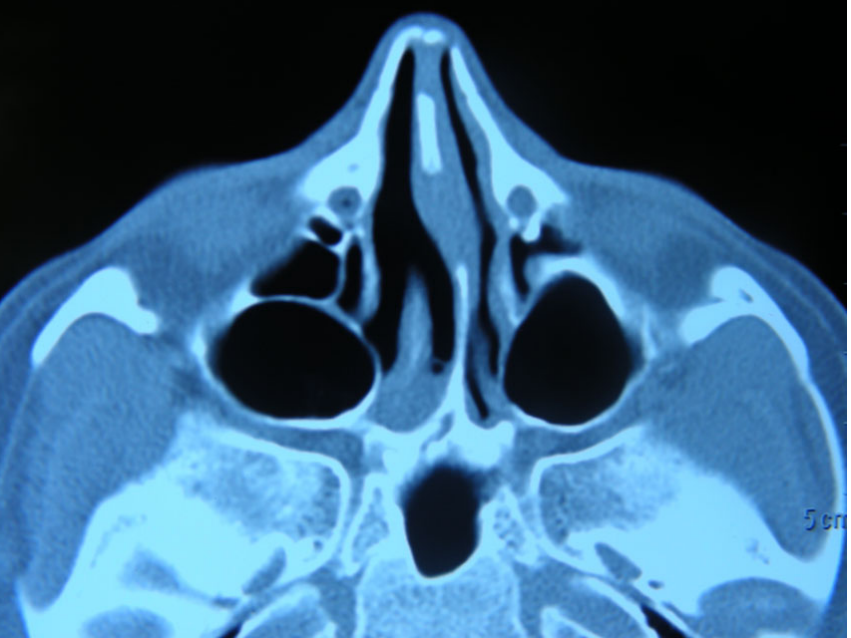
**Bilateral choanal atresia is seen in axial plane computer tomography of the paranasal sinuses**.

A 23-year old-female patient presented to the outpatient clinic with bilateral nasal obstruction and discharge since birth. Endoscopic examination and paranasal sinus tomography revealed bilateral choanal atresia (Figure 1). She did not have any other congenital abnormalities. Her parents reported cyanosis in childhood that worsened during feeding and improved during crying; however, they had not visited a physician. She did not have remarkable complaints during early childhood or adolescence.

**Figure 2:**
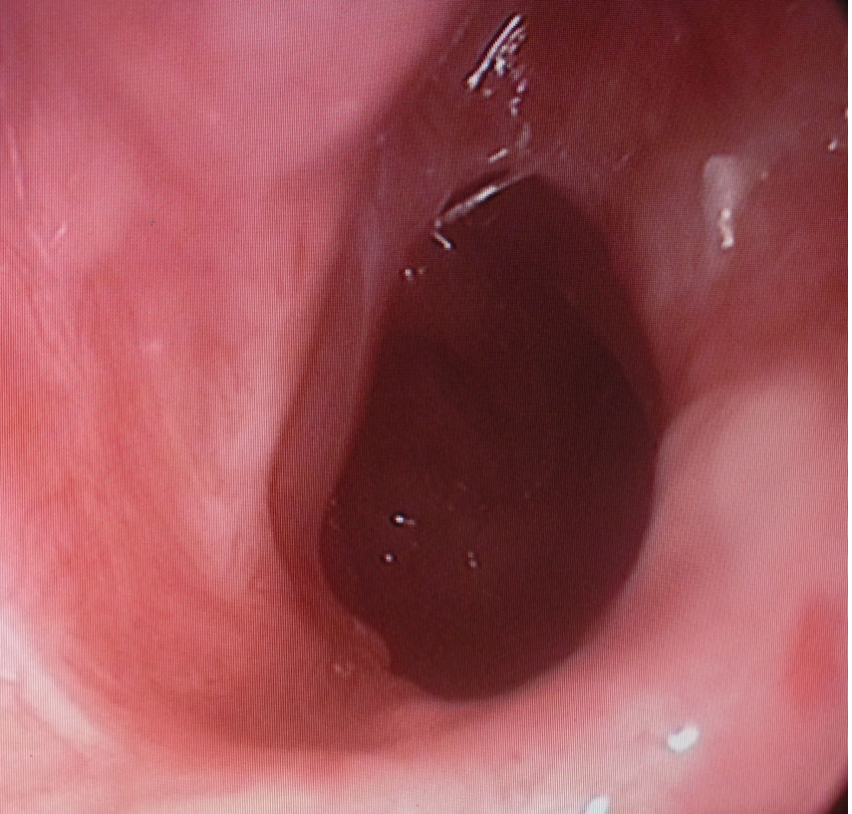
**Left choanal opening 12 months after surgery**.

**Figure 3:**
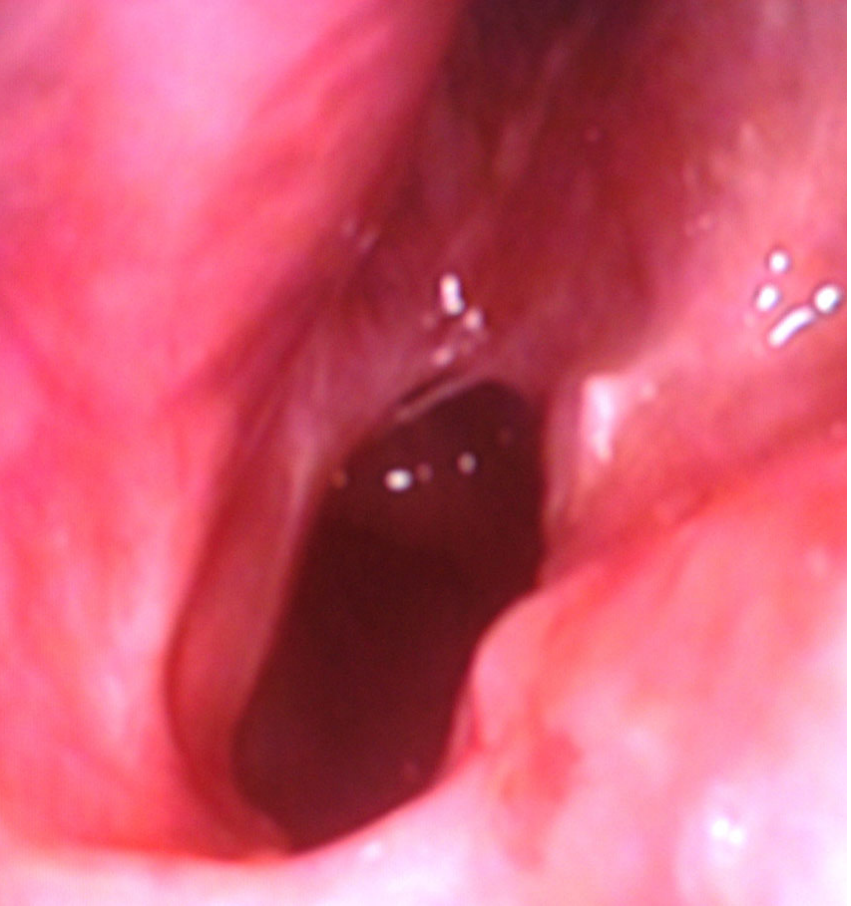
**Right choanal opening 12 months after surgery**.

The patient was operated under general anesthesia, using a 0° 4 mm rigid endoscope. Following local anesthetic infiltration, a mucosal flap was elevated with a sickle-shaped blade. The bony plate was perforated inferomedially with shaver. The orifice was widened with a curette to allow the passage of a number 7.5 endotracheal tube and, to prevent stenosis, cotton soaked with mitomycin-C (1 mg/ml) (Kyowa Hakko Kogyo, Ltd., Tokyo, Japan) was applied topically to the nasopharyngeal orifice for 5 minutes. These procedures were repeated on the contralateral side. No stents were placed. The patient was discharged the next day after surgery and recommended to daily irrigate the nasal cavity with saline. Follow-up evaluation at postoperative 12th month showed that her symptoms improved significantly and, on endoscopic examination, both choanae remained patent (Figure 2,3). Written informed consent was obtained from the patient for publication of this case report and accompanying images.

## Discussion

Choanal atresia occurs as a result of an abnormality in the migration and development of neural crest cells. The frequent asssociation with other congenital abnormalities necessitates thorough evaluation of the patients. Nasal respiration is compulsory during the newborn period, so bilateral choanal atresia is often diagnosed early. The typical clinical picture is cyclic cyanosis and dyspnea that worsens during feeding and improves during crying. There are only three cases of bilateral choanal atresia in adults reported in the literatures [3,5,6].

Panda et al. [3] reported a 22-year-old patient with bilateral choanal atresia. The passage in this patient was established via a transnasal endoscopic approach using a 2.5 mm diamond burr. Subsequently, a number 6 portex cannula was inserted for 6 weeks. Computer tomography of this patient one year after the operation showed that adequate opening was preserved. There were no other congenital abnormalities in this patient.

Yasar and Ozkul [6], reported a 51-year-old patient with bilateral choanal atresia. Using a transnasal endoscopic approach, they raised mucosal flaps and achieved an opening that allowed passage of a number 7.5 portex cannula. The stent was removed 7 days later, and follow-up examination at the 18th month showed that patency was preserved.

El-Sawy et al.[5] reported a 24-year-old patient with bilateral nasal obstruction and total loss of sense of smell and hypogammaglobulinemia. Computer tomography of this patient revealed aplasia of frontal and sphenoid sinuses and hypoplasia of the inferior and middle turbinates. Adequate opening was achieved with transnasal endoscopic approach; however, restenosis requiring a second operation occurred.

In this case report, a 23-year-old patient with bilateral choanal atresia is presented. In our case, we preferred the transnasal endoscopic approach as reported in the previous literatures [3,5,6]. In order to prevent stenosis, Mitomycin-C, previously used by Prasad et al. [7] in 20 patients with choanal atresia without any complication, was applied to the nasopharyngeal opening. Stent was avoided due to the requirement of intense antimicrobial therapy, foreign body reaction and fear of skin necrosis in the columella. Saline irrigation was performed for 12 months after the operation. Regular postoperative controls were made and final examination at the 12th month showed an adequate passage.

## Conclusion

A 23-year-old adult patient with bilateral choanal atresia was operated under endoscopic vision. Follow- up evaluation at postoperative 12th month showed that the opening was preserved.

## Consent section

Written informed consent was obtained from the patient for publication of this case report and accompanying images.

## Competing interests

The authors declare that they have no competing interests.

## Authors' contributions

FA: interpretation of data. HD: conception and design. YSY: conception and design. OO: revising it critically for important intellectual content
